# Genetic Factors Associated with Heading Responses Revealed by Field Evaluation of 274 Barley Accessions for 20 Seasons

**DOI:** 10.1016/j.isci.2020.101146

**Published:** 2020-05-11

**Authors:** Kazuhiro Sato, Makoto Ishii, Kotaro Takahagi, Komaki Inoue, Minami Shimizu, Yukiko Uehara-Yamaguchi, Ryuei Nishii, Keiichi Mochida

**Affiliations:** 1 Institute of Plant Science and Resources, Okayama University, Kurashiki 710-0046, Japan; 2 RIKEN Center for Sustainable Resource Science, Yokohama 230-0045, Japan; 3 Kihara Institute for Biological Research, Yokohama City University, Yokohama 244-0813, Japan; 4 School of Information and Data Sciences, Nagasaki University, Nagasaki 852-8131, Japan; 5 RIKEN Baton Zone Program, Yokohama 244-0813, Japan

**Keywords:** Biological Sciences, Plant Genetics, Plant Biology

## Abstract

Heading time is a key trait in cereals affecting the maturation period for optimal grain filling before harvest. Here, we aimed to understand the factors controlling heading time in barley (*Hordeum vulgare*). We characterized a set of 274 barley accessions collected worldwide by planting them for 20 seasons under different environmental conditions at the same location in Kurashiki, Japan. We examined interactions among accessions, known genetic factors, and an environmental factor to determine the factors controlling heading response. Locally adapted accessions have been selected for genetic factors that stabilize heading responses appropriate for barley cultivation, and these accessions show stable heading responses even under varying environmental conditions. We identified vernalization requirement and *PPD-H1* haplotype as major stabilizing mechanisms of the heading response for regional adaptation in Kurashiki.

## Highlights

• Heading of 274 barley worldwide accessions were evaluated for 20 seasons
• Locally adapted accessions show stable heading responses
• Vernalization requirement and *PPD-H1* haplotype stabilize the heading response

## Graphical Abstract

**Figure:**
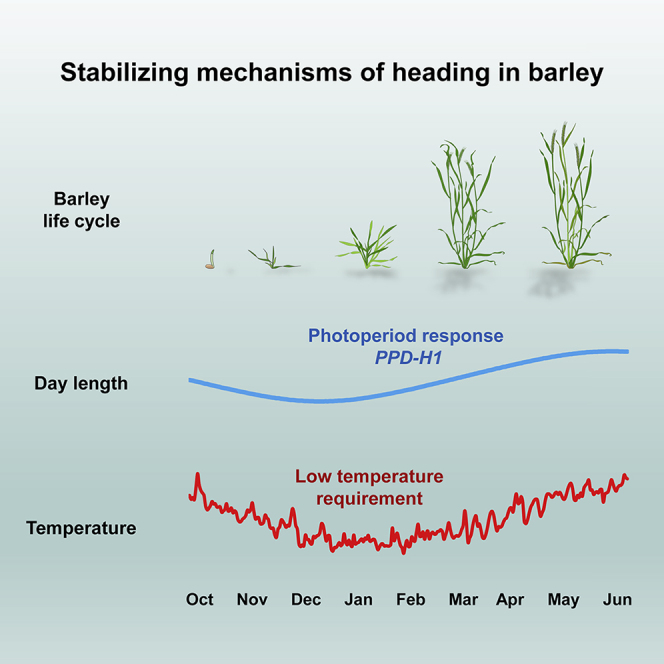

## Introduction

Heading time is the trait when spike emerges from the flag leaf sheath and one of the most important agronomic traits in cereal cultivation. Heading time in barley (*Hordeum vulgare*) is closely related to flowering time and is important in adjusting the maturation period for the most appropriate conditions for grain filling before harvest. Extremely early heading is essential in high-latitude areas such as northern Scandinavia and Alaska, which have short seasons and marginal environments for spring barley cultivation (Faure et al., 2012). Northern European cultivars tend to have longer maturation periods to achieve high yield under longer photoperiod and cooler summer seasons (Jones et al., 2011). East Asian autumn-sown barley cultivars have long growth periods but early heading to avoid the rainy season, which may start at the end of maturity (Ibrahim et al., 2018). Thus, heading time in local barley cultivars reflects adaptation for maturing under the most appropriate conditions for achieving high yield and quality in each area.

Key genetic factors control the heading response in barley. In addition, a low-temperature requirement for flowering, i.e., vernalization, prevents excessive growth and allows escape from cold damage in winter barley (Saisho et al., 2011). A set of *VERNALIZATION* (*VRN*) genes have been identified in barley together with orthologous genes in wheat (*Triticum aestivum*) (Saisho et al., 2011). *VRN-H1* encodes a MADS box transcription factor that promotes flowering by regulating the expression of other genes and promotes the transition from vegetative to reproductive growth (Deng et al., 2015). *VRN-H2* represses flowering in plants that have not been vernalized (Deng et al., 2015). A *VRN-H1/VRN-H2* epistatic model has been proposed to explain the gradation in vernalization requirements among genotypes (Comadran et al., 2012).

Photoperiod (day length) gradually changes from autumn to spring, controlling the timing of flowering in winter-grown barley. *PPD-H1* is the major determinant of the barley photoperiod response and represents a pseudo-response regulator, a class of genes involved in circadian clock function (Turner et al., 2005). *EARLY FLOWERING3* (*ELF3*) is another circadian clock gene that contributes to photoperiod-dependent flowering in plants, with loss-of-function mutants in barley flowering early under noninductive short-day photoperiods (Boden et al., 2014). *HvLUX1*, an ortholog of the *Arabidopsis thaliana* circadian gene *LUX ARRHYTHMO*, controls photoperiod responses in barley (Campoli et al., 2013). *CO1* is a barley homolog of *CONSTANS* (*CO*), which functions in the photoperiodic regulation of flowering in *Arabidopsis* (Mulki and von Korff, 2016). *CEN4* is a barley homolog of *CENTRORADIALIS* (*CEN*) in *Antirrhinum majus* and regulates inflorescence architecture (Comadran et al., 2012). *HvPHYC*, a barley homolog of *PHYTOCHROME C* locus, is also responsible for earliness and is a key factor in controlling long-day flowering in barley (Nishida et al., 2013). A model based on interaction between the vernalization and photoperiod pathways has been proposed for the control of flowering (heading) in barley (Drosse et al., 2013).

Genetic factors responsible for heading may change their quantitative effects depending on plant growth conditions. Spring barley cultivars do not require vernalization, so *VRN* genes may not primarily control heading time in plants growing from spring to summer (Cuesta-Marcos et al., 2015). Day length does not change much in areas at low latitudes; therefore a series of genes controlling photoperiod may not contribute much in these areas (Whittal et al., 2018). Although genetic factors may control barley heading, different environmental conditions exist in crop fields (Cho et al., 2017). Sowing time, nutrition, and moisture can be partly controlled by cultivation practices, but temperature and precipitation after sowing are mostly out of human control (Ibrahim et al., 2018). These environmental conditions may alter the heading time of barley (Cho et al., 2017). Nevertheless, it is known that cultivars well adapted to an area show stable heading dates even under extreme differences in environmental conditions; however, unadapted cultivars may show large deviations from average heading behaviors.

Several methods have been employed to detect genotype-by-environment interactions in field crop performance. Estimation of yield stability across regions and seasons is essential for cultivar release because poor regional yields may restrict the recommended cultivation area, and annual performance data are necessary to demonstrate the stability of economic yield from a cultivar. The classical regression analysis method of Finlay and Wilkinson (1963) is still applicable for detecting deviation from average trait performance, as Gage et al. (2017) recently demonstrated through the Genomes to Fields (G2F) Maize Genotype × Environment (G × E) project to assess the effect of selection on G × E variation and characterize genetic polymorphisms associated with plasticity.

Here, we analyzed datasets of heading response in barley germplasm collected worldwide and grown across 20 seasons at the same location in Kurashiki, Japan. We observed that some cultivars had stable annual heading dates, whereas others showed responses that varied greatly under differing environmental conditions. Our aim was to understand the major genetic and environmental factors controlling heading responses in barley, based on the diverse worldwide barley collection, and to identify accessions showing stable annual heading responses. We also sought to understand the causes of unstable/unadapted heading responses in barley under the growth conditions of Kurashiki to provide information for controlling the heading responses.

## Results

### Genetic Diversity of Barley Accession Was Mainly Clustered by Geographic Origin

**Figure 1:**
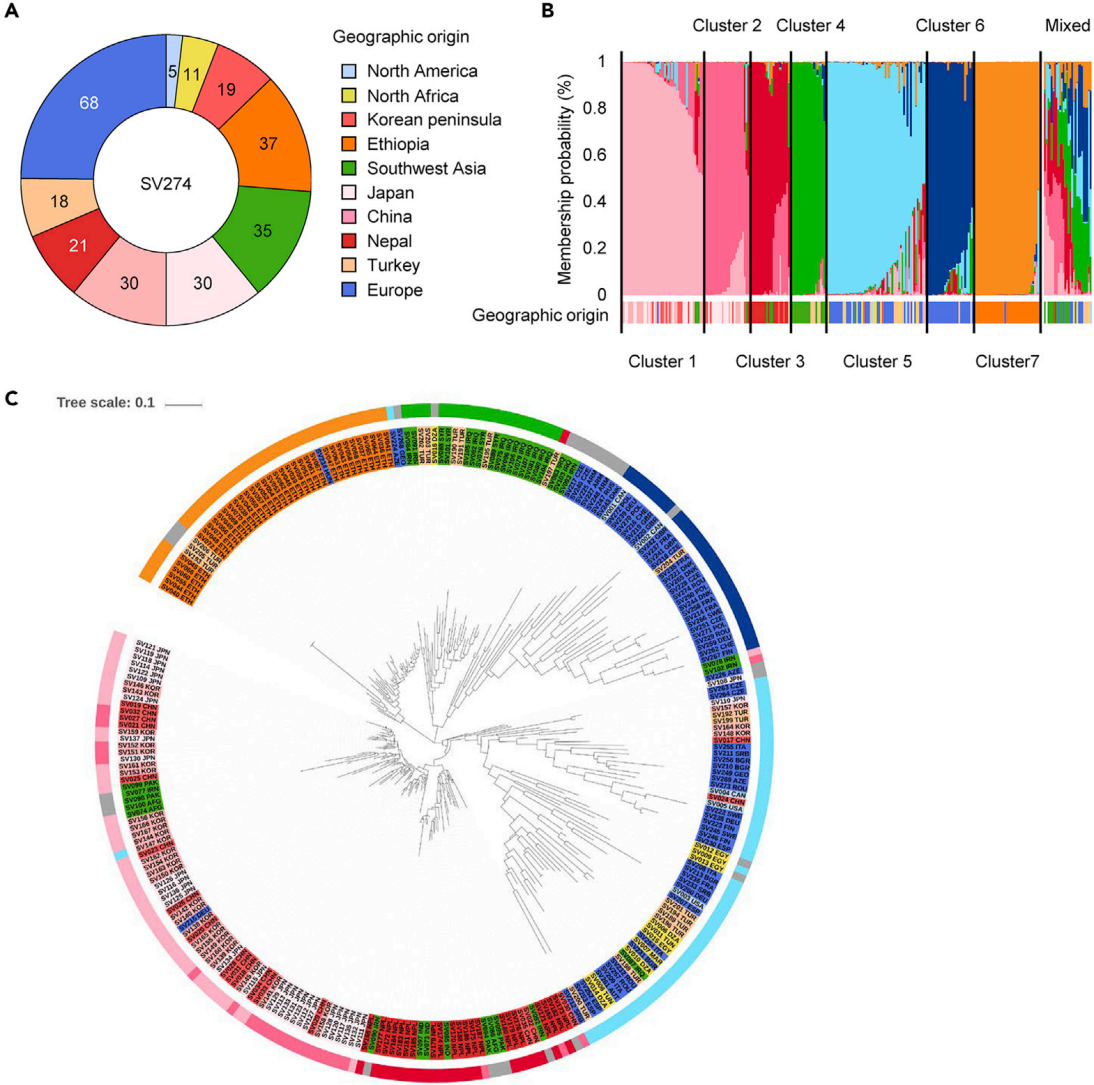
Geographic Diversity and Genetic Structure of the 274 Barley Accessions (A) Geographic distribution of the 274 barley accessions. (B) Population structure of the 274 barley accessions based on 232 SNP markers genotyped by the BOPA SNP Chip. The graph shows the result of Bayesian clustering obtained from a STRUCTURE analysis using *K* = 7. Colors for each bar indicate the probability of corresponding to a specific cluster. Accessions with the probability of belonging to a specific cluster ≥50% are assigned to that cluster. Accessions with probabilities of belonging to any cluster <50% are assigned to the Mixed group. Color codes for each geographic origin used in (A) are provided at the bottom of the graph. (C) Phylogenetic tree of the 274 barley accessions based on 232 SNPs. The 274 accessions are colored according to their origin (inner circle) and genetic clusters (outer circle) defined in (A) and (B), respectively. Scale bar: distance of 0.1.

We estimated genome-wide diversity of 274 barley accessions using the skeletal set of 384 SNP markers derived from BOPA1 (1,536 SNPs) (Close et al., 2009). Of these 384 SNPs, 232 produced marker genotypes without missing data. The accessions had diverse geographic origins (10 areas), with the most (68) accessions from Europe (Figure 1A). Bayesian clustering obtained from STRUCTURE analysis (Pritchard et al., 2000) indicated the number of clusters as *K* = 7 for the 274 accessions (Figure 1B). Clusters 1 to 3 mostly included East Asian accessions from China, the Korean Peninsula, Nepal, and Japan. Cluster 4 was composed of Southwest Asian and North African accessions. Cluster 5 was a mixture, with the majority of accessions from Europe. Cluster 6 was mainly composed of accessions from Europe. Cluster 7 was composed mostly of Ethiopian accessions. A phylogenetic tree based on marker dissimilarity showed that genetic distance and clustering partly agreed with that obtained by Bayesian clustering, indicating that the materials used in the analysis have specific haplotypes linked to geographic origins (Figure 1C).

### Genetic×Environment Interactions Control Heading Responses

We built linear regression models of DHS in 257 accessions observed for 20 seasons (*n* = 5,140) on genetic factors, an environmental factor (season), and their interactions (Table 1). All sets of factors showed highly significant *F*-values at the 0.1% level. Among the main (single) factors, accession showed the largest *R*^*2*^ (0.66). The best model with adjusted *R*^*2*^ (0.89) with high *F*-value was obtained using accession + season (Table 1), indicating that the interaction accession + season plays an important role in estimating DHS.

**Table 1:** Linear Regression Analysis of Days to Heading from Sowing on Genetic and Environmental Factors

Factor	d.f. (Regression)	d.f. (Error)	Adjusted *R*^*2*^	*F*-value
**Genetic Factors**				

Accession	256	4,883	0.66	39.4^a^
Origin (country of origin)	36	5,103	0.32	68.6^a^
Population cluster	7	5,132	0.11	91.2^a^
Growth habit (spring/winter growth habit)	1	5,138	0.01	48.5^a^
Kernel row (two/six row)	1	5,138	<0.01	9.1^a^

**Environmental Factor**				

Season (growing season)	19	5,120	0.22	76.0^a^

**Genetic Factor + Environmental Factor**				

Accession + season	275	4,864	0.89	148.4^a^
Origin + season	55	5,084	0.54	111.2^a^
Population cluster + season	26	5,113	0.33	97.2^a^
Growth habit + season	20	5,119	0.23	76.2^a^
Kernel row + season	20	5,119	0.22	73.0^a^

See also Figures S1–S4.

a Significant at the 0.1% level.

### Variation in Heading Response Was Different among Geographical Areas of Origin

**Figure 2:**
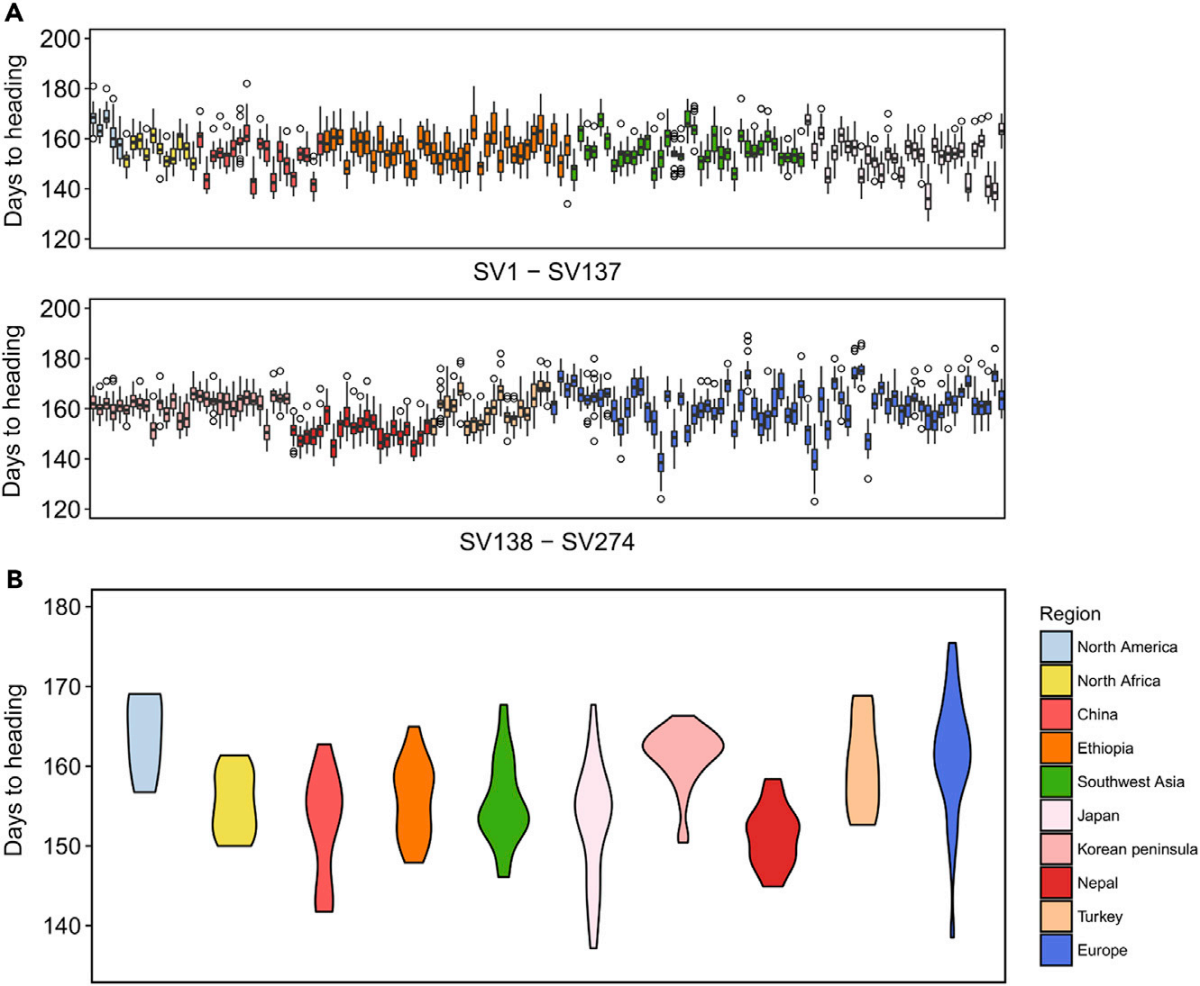
Geographic Origins Show Patterns in Heading Date across 274 Barley Accessions (A) Boxplots show days from sowing to heading for each accession colored by its geographic origin based on the 20-season field test at IPSR, Okayama University. (B) Violin plot of variation for 20-season data in each region. See also Figures S1 and S2, Tables S1 and S2.

Each accession showed variation in DHS across 20 seasons (Figure 2A). The mean DHS across 20 seasons for each accession ranged from 137 to 175, with standard deviations ranging from 3.1 to 8.0 (Table S1). Variation in DHS was different among the geographical areas of origin (Figure 2B). Accessions from Japan and Europe showed wide variation with a small number of accessions exhibiting extremely short or long DHS. North American and Turkish accessions displayed longer DHS with smaller, less-extreme distributions.

To characterize the DHS response in each accession, we analyzed linear regression of DHS across 20 seasons for each accession on the mean DHS of 257 accessions using the method of Finlay and Wilkinson (1963) (Table S2 and Figure S1). The regression coefficients ranged from 0.28 (SV135) to 1.53 (SV068) (Table S2). Coefficients of multiple determination (*R*^*2*^) were generally high; however, these were lower for some accessions, especially those with low regression coefficients (SV135, SV167, SV136, and SV129). By contrast, accessions with high linear regression coefficients showed high *R*^*2*^, indicating that DHS of these accessions respond well to environmental conditions. Correlation coefficients between Finlay-Wilkinson linear regression coefficients and other parameters (Table S1) were significantly high or low for standard deviation (*r* = 0.735), range (*r* = 0.524), vernalization requirement (*r* = −0.429), and longitude (*r* = −0.390). These results suggest that the accessions with high Finlay-Wilkinson linear regression coefficients might have greater variation and wider range of DHS. They also tended to have lower vernalization requirements (spring growth habit) and were originated from lower-longitude areas. Many of the Japanese accessions were included among those showing low linear regression coefficients (Figure S2), whereas a number of Ethiopian accessions were included among those showing high linear regression coefficients (Figure S2).

### Deviated Heading across Accessions Made Disagreement from the Average Season

**Figure 3:**
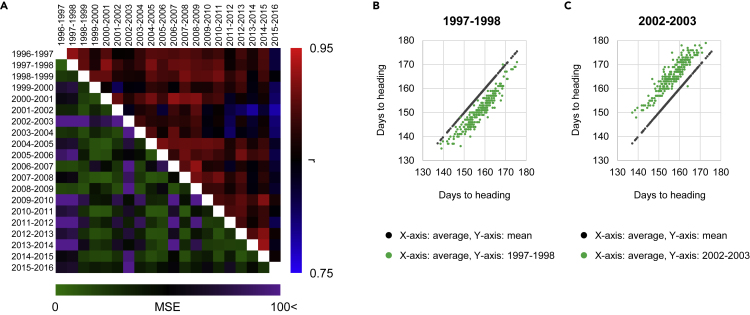
Seasonal Plasticity of Heading Date Correlates with the Deviation of 274 Barley Accessions (A) Heatmap showing pairwise Pearson correlation coefficients (*r*) and mean square errors (MSE) of days from sowing to heading in the 274 barley accessions between seasons. (B and C) Scatterplots showing distribution of days from sowing to heading in the 274 barley accessions in the (B) 1997–1998 and (C) 2002–2003 growing seasons (green dots) compared with mean days from sowing to heading in the 20-season field test (black dots). See also Figures S3 and S4, Tables S3 and S4.

The mean DHS across all accessions in each season varied from 150.5 to 166.0 days with standard deviations from 6.0 to 9.7 (Table S3). The phenotypic plasticity of DHS in each season is presented as pairwise Pearson correlation coefficients (*r*) and mean square error (MSE) in Figure 3A. The value of *r* was high in the 1997–1998 season, but most accessions showed delayed heading, except for a few accessions with DHS close to the mean DHS (Figure 3B). The lowest MSE was observed in the 2002–2003 season when accessions headed earlier than mean (Figure 3C). The linear regression of DHS in 257 accessions in each season on the mean DHS across 20 seasons was analyzed by the method of Finlay and Wilkinson (1963) (Table S4 and Figures S3 and S4). The linear regression coefficient ranged from 0.81 to 1.32 with *R*^*2*^ from 0.89 to 0.96 (Table S4), indicating a high goodness of fit of DHS in each season with the seasonal mean, but with differences among seasons (Figure S4). Correlation analysis between DHS-related parameters in Table S3 and Finlay-Wilkinson linear regression coefficients in Table S4 indicated significantly high correlation coefficients for standard deviation (0.988) and range (0.805). These results indicated that the similarity of the annual DHS to that in an average season was influenced by the variation of the DHS of accessions in each season.

### Heading-Related Genes Control Heading Response

**Figure 4:**
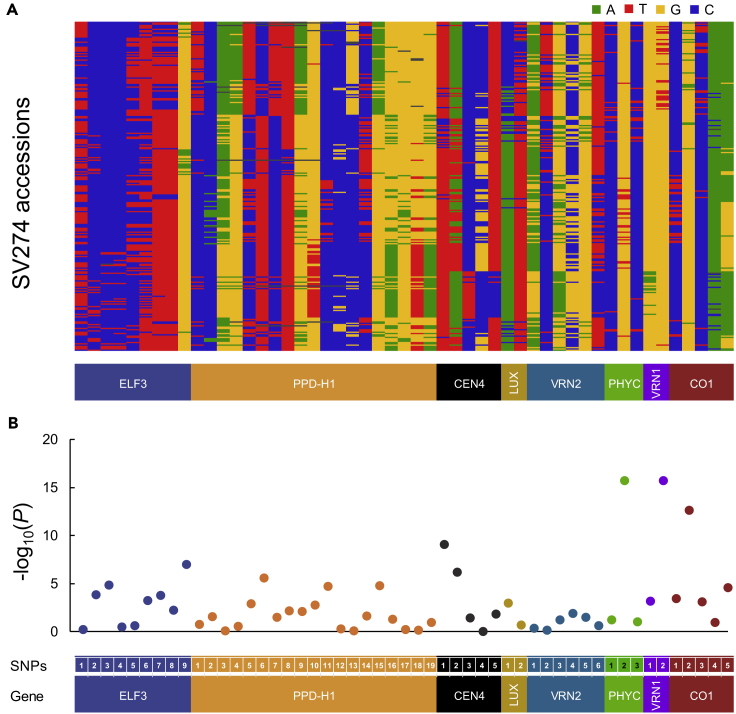
SNPs in Eight Flowering-Related Genes of Barley Affect Heading Date Prediction (A) Nucleotide diversity in 51 SNPs in eight flowering-related genes among the 274 barley accessions based on sequence comparison with the cv. Morex reference genome (Mascher et al., 2017). (B) Scatterplot representing the significance of each SNP affecting heading date prediction. *SNF2P* was used for estimating the genotype of *VRN2* (Cuesta-Marcos et al., 2010). y axis shows the value of –log10-scaled p value for testing H_0: no association between tested SNP and trait. x axis shows physical order of the SNP within genes that are separated by different colors. See also Tables S4 and S5.

To estimate the effects of known flowering-related genes on the heading response in barley, we genotyped 84 SNP alleles derived from eight genes or markers by amplicon sequencing-based genotyping (Table S5). Of these, 51 alleles were identified through variable selection in a linear regression analysis using DHS data for 234 accessions (Figure 4A). We estimated the contribution of each SNP to the heading response using linear regression analysis (Figure 4B), with the p value of regression coefficient plotted by −log 10 (p value). The haplotypes in Figure 4A are ordered based on the clusters (from 1 to 7) developed by the genome-wide 384-SNP genotyping platform shown in Figure 1B. Some SNPs (*PHYC*-#2, *VRN1*-#2, and *CO1*-#2) showed more than 10 -log_10_(*P*) for their contribution to heading. However, alleles of these SNPs were not concentrated in specific accessions, as shown in Figure 4A.

**Figure 5:**
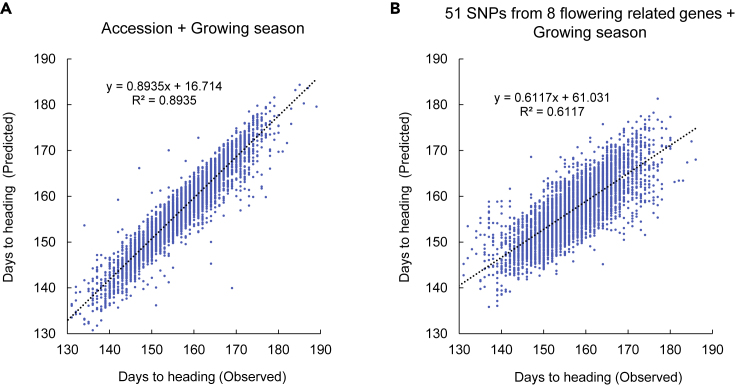
Flowering-Related Genes Predict Part of the Phenotypes for Heading Date in the Barley Accessions (A and B) Scatterplots show the results of linear regression analysis of observed and predicted days to heading from sowing for 234 accessions without missing values across 20 seasons based on the model of (A) accession and growth season (*R*^2^ = 0.8935; *y* = 0.8935*x* + 16.714) and (B) 51 SNPs from eight flowering-related genes and growth season (*R*^*2*^ = 0.6117; *y* = 0.6117*x* + 61.031). See also Tables S2–S6.

We also estimated the contribution of the 51 SNP alleles derived from the eight flowering-related genes on the power of prediction of DHS (Figure 5). We used the 234 accessions having datasets without missing values for DHS across 20 seasons for linear regression analysis. Predicted values based on the regression (*y* = 0.8935*x*+16.714) were plotted against the observed values, showing a high goodness of fit with *R*^*2*^ = 0.8935 (Figure 5A). We conducted a similar prediction using the 51 SNPs derived from eight flowering-related genes with the linear regression (*y* = 0.6117*x* + 61.031) (Figure 5B). A moderate *R*^*2*^ value (0.6117) was obtained from the plots of predicted and observed values, but this was much lower than that obtained using the observed DHS across 20 seasons (Figure 5A).

We also used the contributions of the 51 SNP alleles as independent variables in multiple regression analysis to estimate DHS as a dependent variable (Table S6). The multiple regression coefficient *R*^*2*^ was 0.531. Of the 51, two SNPs from *PPD-H1* (29126802 and 29126824) showed significant standardized partial regression coefficients. The recalculated coefficient of multiple determination only with these two SNPs showed *R*^*2*^ = 0.207 with *F*-value = 32.39 (*p* = 0.00), indicating that these SNPs significantly contributed to the accession responses to seasonal conditions.

## Discussion

Several models of flowering have been proposed in *Arabidopsis* and cereals using mutants (Bäurle and Dean, 2006, Yoshida and Nagato, 2011, Drosse et al., 2013). These models include detailed relationships among genetic components to control flowering. However, the quantitative contribution of each component is not clear. Actual flowering time is influenced by environmental conditions (Cho et al., 2017, Matsubara, 2018, Li et al., 2018), which are difficult to control by cultivation practices. Our analysis demonstrated that repeated evaluation across multiple seasons is an efficient, albeit time-consuming, method for estimating the contribution of genetic factors and their interactions with the environment. As shown in Table 1, heading response was mainly affected by genetic factors, whereas environmental factors mostly contributed through interaction with genetic factors. These factors are examined in the following sections.

### Genetic Factors Affect Heading Behavior in Barley

In this study, we used data for DHS observed at one location in Kurashiki, Japan. This location has the advantage of good growth conditions for all winter and spring habit barley accessions with early to late growth. For this reason, we grew most of the barley materials collected worldwide in an *ex situ* gene bank of barley at the Kurashiki campus of Okayama University. As shown in Figure 1A, we chose a set of 274 accessions from the barley collection at Okayama University having diverse origins and differentiated haplotypes, which might represent the diversity of the heading responses in barley.

Of the 274 accessions used in our study, 103 accessions had winter habits. The spring/winter growth habit showed a significant contribution in the regression analysis in Table 1. The significant correlation coefficient (*r* = 0.429) between the vernalization requirement in Table S1 and the linear regression coefficients in Table S2 also indicated that accessions with higher vernalization requirement tend to have stable DHS and that spring/winter growth habit might be one of the factors stabilizing the heading response. The ancestral wild form of cultivated barley (*Hordeum vulgare* subsp. *spontaneum*) has a winter habit, flowering in spring and maturing in early summer (von Bothmer et al., 2003). Pourkheirandish et al. (2015) estimate that two domestication events occurred in this wild barley at North and South Levant, with the resulting cultivated barleys distributed to Asia and Europe, respectively. Spring barley has mutated from these domesticated winter barleys at the gene *VRN1*, *VRN2*, or *VRN3*. Substitution of a spring allele at any of the *VRN* loci is sufficient to eliminate the vernalization requirement (Cuesta-Marcos et al., 2015). These spring barleys might be adapted to cultivation in high-latitude areas where winters are too cold for winter barley to grow. The multiple mutation haplotypes *VRN1/VRN3* and *VRN1/VRN2/VRN3* have also developed during the adaptation processes (von Bothmer et al., 2003). Many of the accessions with the lowest linear regression coefficient to the season mean (Table S2) show winter habit, e.g., SV135 (0.28) collected from western Japan. These results suggest that vernalization requirement is a possible factor stabilizing heading response in temperate winter barley-growing areas, e.g., Kurashiki located in western Japan.

Many of the genes related to flowering control photoperiod responses (Brambilla et al., 2017). We observed significant correlation coefficients between standard deviation (*r* = 0.735), range (*r* = 0.524), longitude (*r* = −0.390), and the Finlay-Wilkinson linear regression coefficient of DHS for each accession on means of 20 seasons (Table S2). The 20 accessions with the highest linear regression coefficients included nine Ethiopian accessions (of the 37 Ethiopian accessions). As Ethiopia is located close to the equator (also lower longitude), seasonal changes in day length are small and plants may therefore not need strong photoperiodic responses. Milner et al. (2019) genotyped 22,000 world barley accessions preserved in the German gene bank and reported that Ethiopian accessions were located at a different vertex of a triangle from other European and East Asian vertices. We also identified that the haplotype of Ethiopian accessions was different from those of North African or South East Asian accessions (Figure 1C) and might have developed through a specific evolutionary process. Many of the flowering-related genes used for SNP genotyping (Figure 4) have orthologous genes in other plant species (Higgins et al., 2010). As barley shows the widest worldwide distribution among cereal crops, some of the distribution and adaptation processes of barley might be independent from those of other crops. Particularly, the ancestral haplotypes of domesticated barley might be quite different from those of rice or maize, with the place of domestication and cultivation conditions during the distribution process causing specific selection pressures (Pankin et al., 2018). Barley distribution to Ethiopia was a man-made event because no direct domestication occurred in the area and accessions from other parts of the world show no haplotype similarity due to a long period of isolation from Ethiopian landraces (Orabi et al., 2007).

### Genetic×Environment Interactions Influence Heading Behavior in Barley

DHS in most seasons showed high correlation (*r*) with mean DHS values across 20 seasons (Figure 3 and Table S4). However, linear regression coefficients for DHS in each season against the mean DHS from 20 seasons varied from 0.81 (2005–2006) to 1.32 (2009–2010) (Table S4). Detailed observation of plots in 2005–2006 showed that early-heading accessions headed earlier than mean but late-heading accessions headed at the expected time (Figures S3 and S4). In 2009–2010, early-heading accessions headed at the expected time but late-heading accessions headed later than mean. If we apply the range of mean DHS across 20 seasons (137–175 days), the linear regressions of *y* = 0.81*x*+31.68 (2005–2006) and *y* = 1.32*x*-45.79 (2009–2010) in Table S4 result in ranges of 143–173 and 135–185 days, respectively, indicating that the range of 2005–2006 is 20 days shorter than that in 2009–2010. These two extreme seasons indicate that environmental conditions may influence the heading dates in accessions; however, a group of accessions with close heading dates respond similarly. These results and the significant main effect of the environmental factor (season) shown in Table 1 indicate that the environmental factor influenced unadapted accessions, causing deviations from mean season; however, DHS in adapted accessions did not change much from that in the mean season. Overall, the deviated seasonal responses of DHS were mainly caused by the accessions from alien origins.

### Key Genetic Factors Showing Specific Contribution to Heading Behavior in Temperate Winter Barley-Growing Areas

We used 51 SNPs from eight flowering-related genes to estimate their contributions to DHS stability in western Japan, although these were not the only candidates for controlling heading, as is apparent from the comparison of Figure 5A (Accessions + Growing seasons) with Figure 5B (51SNPs + Growing seasons). Multiple regression analysis (Table S6) revealed that two of the SNPs from *PPD-H1* made a significant contribution to DHS stability. These results indicate that *PPD-H1* is a major factor contributing to DHS stability in Kurashiki, located in western Japan. Combining all the above information, the stability of heading in Kurashiki is mainly achieved by the combination of vernalization requirement and *PPD-H1* haplotypes. We assume that barley haplotypes without these two genetic components may show deviations from the mean DHS of all barley accessions in the temperate winter barley cultivation areas like Kurashiki. Thus, higher levels of vernalization requirement (winter type) and 29126802 (C/T) and 29126824 (G/C) SNP genotypes of *PPD-H1* gene may give lower Finlay-Wilkinson linear regression and give higher stability of heading response in Kurashiki. The information obtained in this study may contribute to select the haplotypes adapted for the growing area and provide useful information for the candidate genetic factors to control heading responses in barley breeding programs.

### Limitations of the Study

The present study has been conducted in one location in Japan, and the application of the results may be restricted to the temperate winter barley-growing regions. We identified two key genetic factors stabilize the barley heading reaction. However, there may be other genetic factors that are not included in the present study. The situation may be different especially in other growing conditions, e.g., long day length spring-sown barley areas like Scandinavian countries.

### Resource Availability

#### Lead Contact

Further information and requests for resources should be directed to and will be fulfilled by the Lead Contact, Kazuhiro Sato (kazsato@okayama-u.ac.jp).

#### Materials Availability

Barley seeds used in this study were available from the National BioResource Project-Barley, Japan (www.nbrp.jp).

#### Data and Code Availability

The datasets used and analyzed during this study are available from the Lead Contact upon reasonable request.

## Methods

All methods can be found in the accompanying Transparent Methods supplemental file.

## Supplemental Information

Document S1. Transparent Methods, Figures S1–S4, and Tables S3–S6
