# Supplementary material for: Genetic Factors Associated with Heading Responses Revealed by Field Evaluation of 274 Barley Accessions for 20 Seasons
Source: iScience. 2020 May 11;23(6):101146. doi: 10.1016/j.isci.2020.101146 (PMC7251784; doi:10.1016/j.isci.2020.101146)
Supplement: Document S1. Transparent Methods, Figures S1–S4, and Tables S3–S6 [file mmc1.pdf]

iScience, Volume *23*

Supplemental Information

Genetic Factors Associated with Heading Responses Revealed by Field Evaluation of 274 Barley Accessions for 20 Seasons

Kazuhiro Sato, Makoto Ishii, Kotaro Takahagi, Komaki Inoue, Minami Shimizu, Yukiko Uehara-Yamaguchi, Ryuei Nishii, and Keiichi Mochida

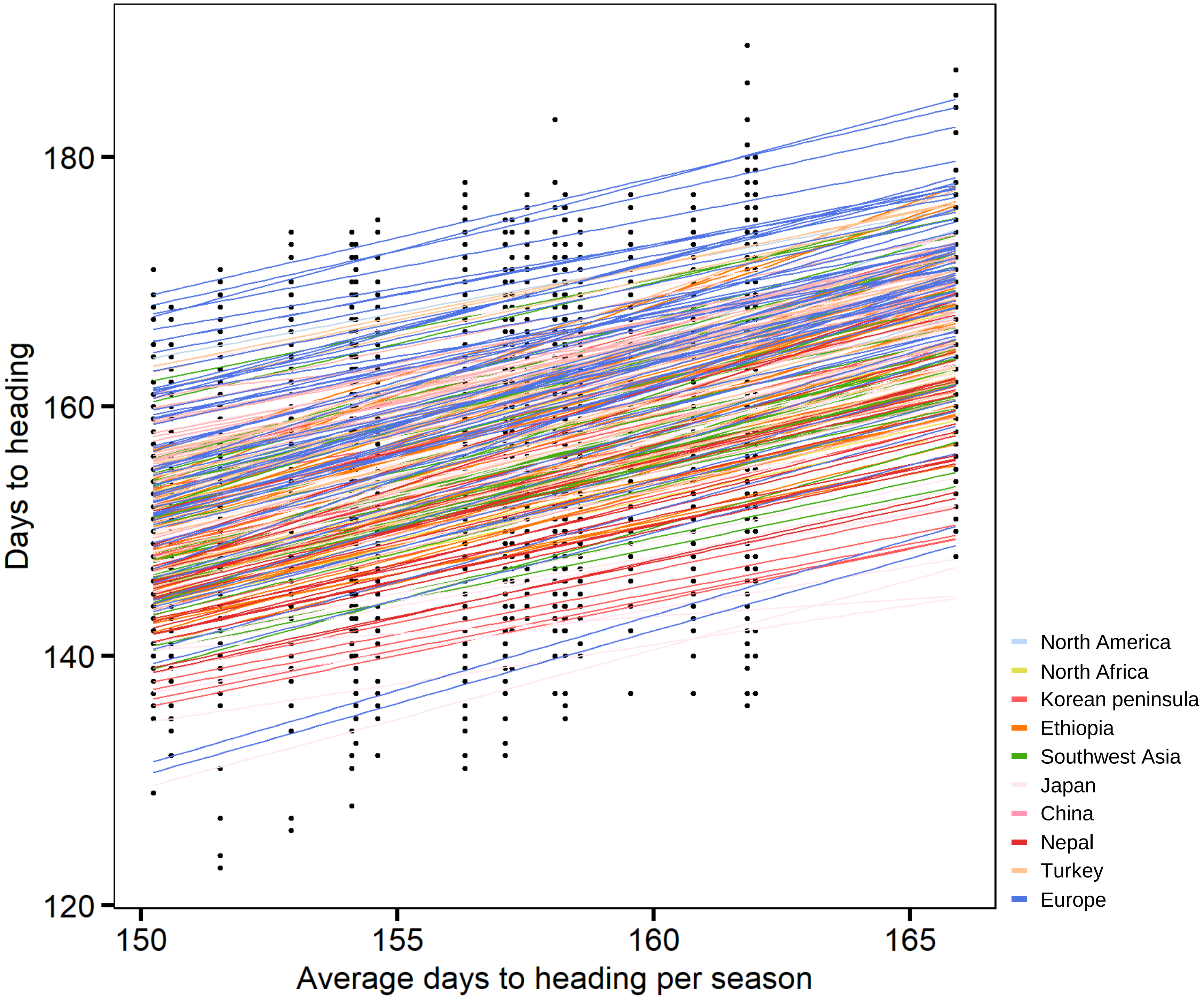

Figure S1. Linear regression fit between means of DHS for the 257 accessions on means of DHS for each of the 20 seasons, Related to Table 1 and Figure 2.

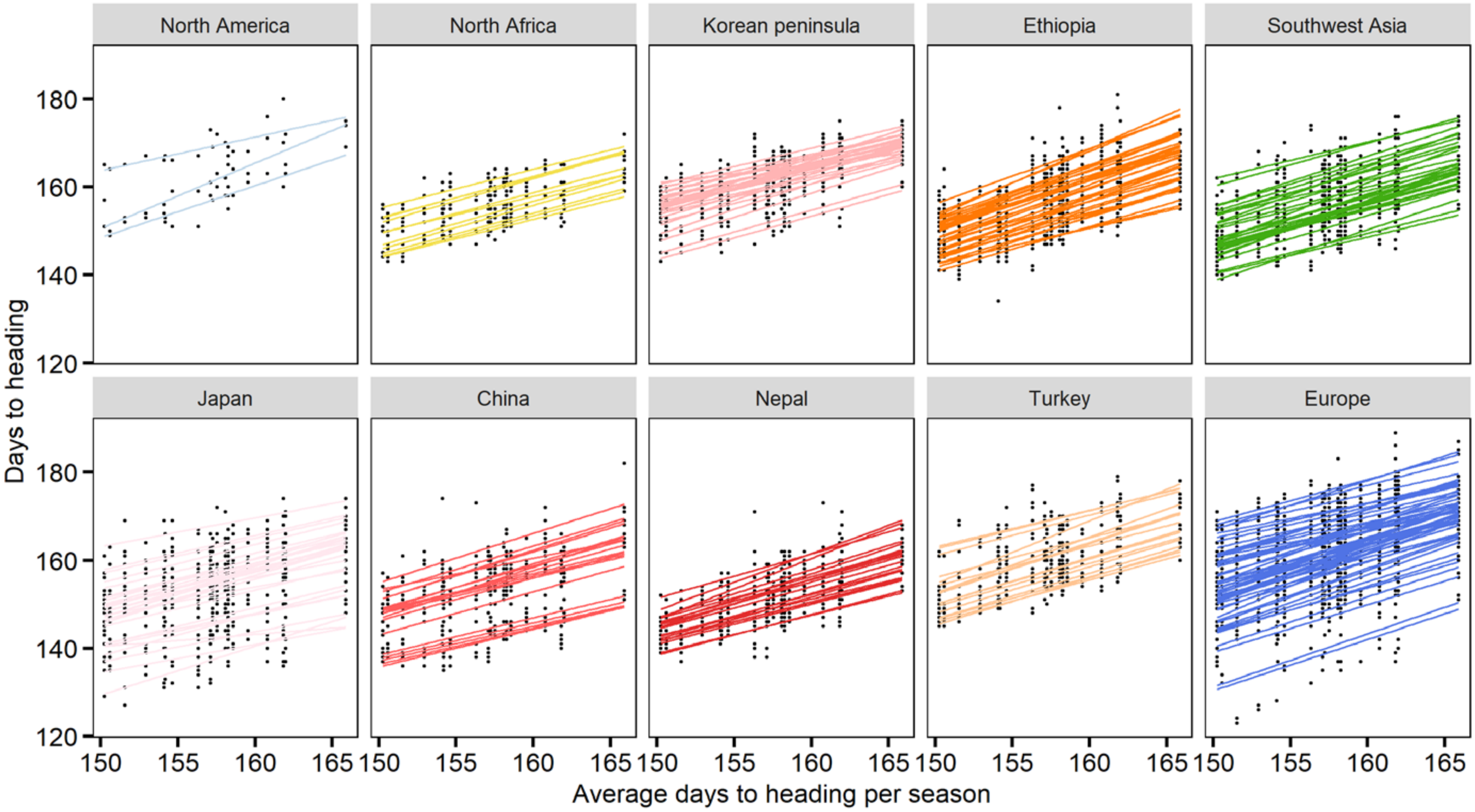

Figure S2. Linear regression fit between means of DHS for the accessions on means of DHS for each of the 20 seasons faceted in geographic origins of the accessions, related to Table 1 and Figure 2.

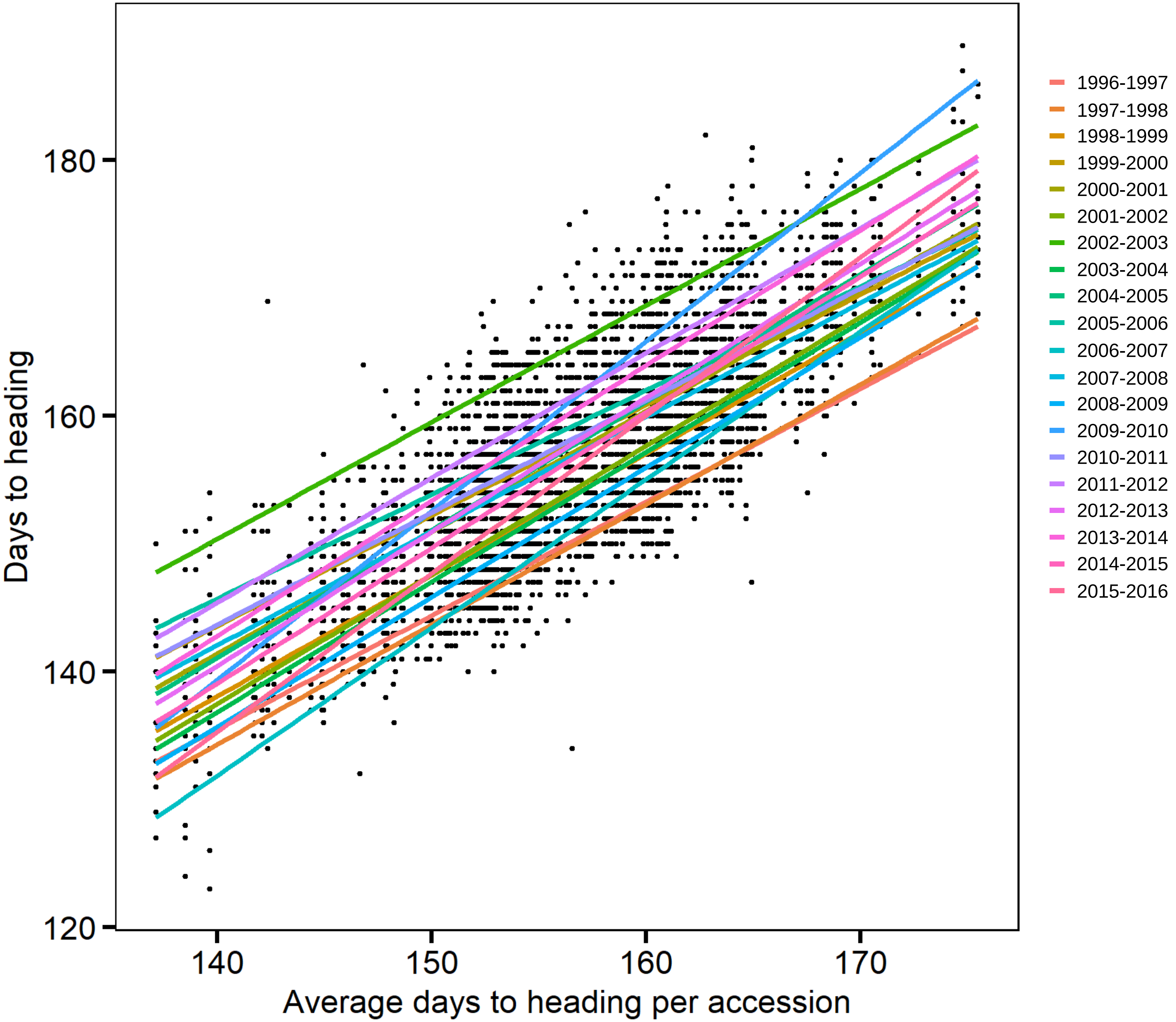

Figure S3. Linear regression fit between means of DHS for each season on the accession means of DHS across the 20 seasons, Related to Table 1 and Figure 3.

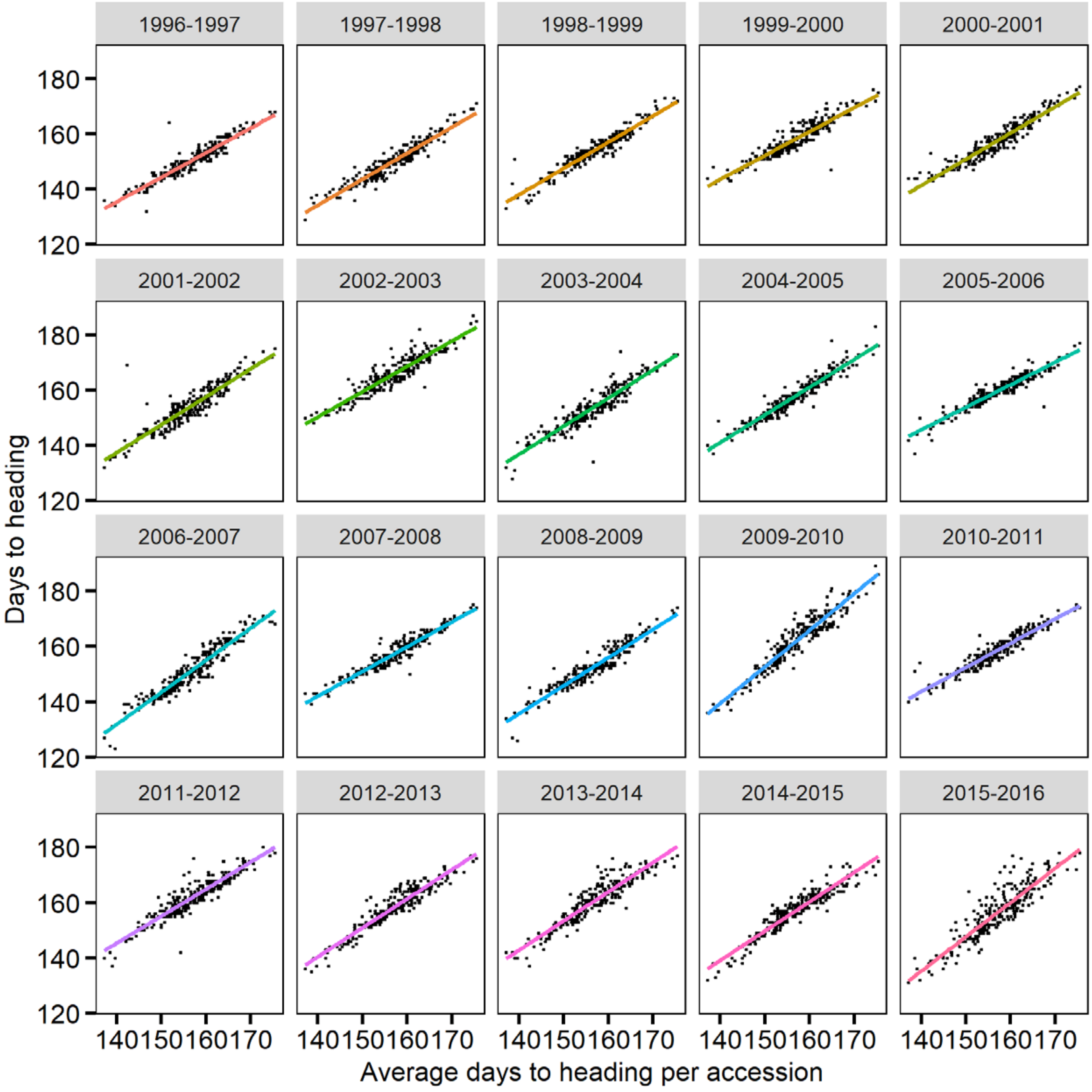

Figure S4. Linear regression fit between means of DHS for each season on the accession means of DHS across the 20 seasons faceted in each season, Related to Table 1 and Figure 3.

Table S3: Basic statistics on the years for the mesurements of days to heading, Related to Figures 3 and 5.

Season	Average	SD	CV	n	Earliest	Latest	Range
1996-1997	150.8	6.5	0.04	273	132	168	36
1997-1998	150.5	6.9	0.05	271	129	171	42
1998-1999	154.4	6.8	0.04	274	133	173	40
1999-2000	158.5	6.5	0.04	272	142	176	34
2000-2001	157.7	7.0	0.04	274	143	177	34
2001-2002	154.9	7.7	0.05	274	132	175	43
2002-2003	166.0	7.0	0.04	266	148	187	39
2003-2004	154.4	7.7	0.05	273	128	174	46
2004-2005	158.4	7.3	0.05	273	137	183	46
2005-2006	159.8	6.0	0.04	274	137	177	40
2006-2007	151.8	8.5	0.06	274	123	171	48
2007-2008	157.5	6.6	0.04	274	139	177	38
2008-2009	153.4	7.6	0.05	274	126	174	48
2009-2010	162.3	9.7	0.06	274	136	189	53
2010-2011	158.8	6.4	0.04	274	140	175	35
2011-2012	162.4	7.4	0.05	274	137	180	43
2012-2013	158.6	7.7	0.05	274	135	177	42
2013-2014	161.0	8.0	0.05	274	137	177	40
2014-2015	157.4	7.8	0.05	274	132	175	43
2015-2016	156.5	9.7	0.06	271	131	178	47

Table S4: Linear regression of days to heading in 257 accessions each season against the mean days to heading across 20 seasons, Related to Figures 3 and 5.

Season	Intercept	Coefficient	Multiple correlation coefficient
1996-1997	11.11	0.89	0.95
1997-1998	2.71	0.94	0.95
1998-1999	5.14	0.95	0.95
1999-2000	22.53	0.86	0.93
2000-2001	8.58	0.95	0.95
2001-2002	-3.74	1.01	0.91
2002-2003	22.42	0.91	0.92
2003-2004	-5.41	1.02	0.93
2004-2005	0.98	1.00	0.96
2005-2006	31.68	0.81	0.94
2006-2007	-30.35	1.16	0.95
2007-2008	16.94	0.89	0.95
2008-2009	-6.60	1.02	0.95
2009-2010	-45.79	1.32	0.95
2010-2011	20.92	0.88	0.95
2011-2012	8.61	0.98	0.92
2012-2013	-6.42	1.05	0.95
2013-2014	-5.43	1.06	0.92
2014-2015	-9.53	1.06	0.95
2015-2016	-38.36	1.24	0.89

Table S5: PCR primers used for amplicon sequencing-based genotyping, Related to Figures 4 and 5.

	Genes	Forward primer F (Oligo DNA seqeucne 5'->3')	Reverse primer (Oligo DNA seqeunce 5'→3')	Reference
VRN-H1	BM5A	BM5.09F (TGGCGAGAAAAAATGATTTGGGGA)	BM5.94R (CGTCCTAACCCTTCCACTTG)	Arifuzzaman et al. 2016
	BM5A (Strider)	BM5.42F (GAAAGCTCTACGAGTTCTCCAC)	BM5.95R (CTAGACCGACAACACATGCAAG)	
	BM5A (Maskin)	BM5.09F (TGGCGAGAAAAAATGATTTGGGGA)	BM5.67R (CTACGCCGAGCACAGAAAGC)	
	BM5A (Albacete)	BM5.42F (GAAAGCTCTACGAGTTCTCCAC)	BM5.59R (CAGAGATGTGGTTTTCACGTTAG)	
	BM5A (Morex)	BM5.42F (GAAAGCTCTACGAGTTCTCCAC)	BM5.59R (CAGAGATGTGGTTTTCACGTTAG)	
	BM5A (OWB-D)	BM5.42F (GAAAGCTCTACGAGTTCTCCAC)	BM5.86R (TCCCCCATTCTCGTCAAAAAGC)	
	BM5A (Triumph)	BM5.42F (GAAAGCTCTACGAGTTCTCCAC)	BM5.43R (TTCTGCATAAGAGTAGCGCTCAT)	
PPD-H1	HvPRR7	HvPRR7.05F (GATGGATTCAAAGGCAAGGAG)	HvPRR7.08R (CGAGCTCCCAATGATCCATG)	
ELF3	HvELF3	ELF3.01F (TGTCAGAGAAAGGCCTAAGAGA)	ELF3.02R (GCTCAAACACTTGGACAGCA)	
LUX1	HvLUX	LUX1.F (GCTCGATTGGTGTTGCTAGG)	LUX1.R (GAGCAGAGAGCAGAGCATCC)	
CEN	HvCEN	CEN.F (TCCTCTCATCTCCAGCCATC)	CEN.R (TGCACGTACACTGGTTCACA)	
PhyC	HvPHYC	Ex1seq_1f (CCCGTCCTTCTCCACAAAAG)	Ex1seq_1r (GAGCCACAGAGGCTGATAGG)	Pankin et al. 2014
PhyC	HvPHYC	Ex1seq_2f (ACTACCCGGCAACTGACATC)	Ex1seq_2r (ACAGAATCACCCTCCACGAG)	
CO1	HvCO1	HvCO1_F1 (TCCAACGGCACCGTTTATGA)	HvCO1_R2 (CGACGTTTCACACTTTCACTTGC)	Stracke et al. 2008
CO1	HvCO1	HvCO1_F4 (TTGGTGCAAGTGAAAGTGTGAA)	HvCO1_R3 (TGTCAGATAGGGCCGCAGTT)	
CEN	HvCEN	CEN_F1 (TTTGGAAGGGAGGTGGTGAG)	CEN_R1 (GAAGTAGACGGCAGCGACAG)	Comadran et al. 2012

Table S6: **Multiple regression analysis of Finlay-Wilkinson linear regression coefficients in Table S3 on 51 SNPs from flowering related genes. A total of 234 accessions without missing data were used, Related to Figures 4 and 5.**

Flowering related gene	SNP (independent valuable)	Standardized partial regression	*F*	*P*	
ELF3	556902172	0.1859	0.0990	0.7533	
ELF3	556902247	-0.0500	0.0825	0.7742	
ELF3	556902476	-0.1078	0.6005	0.4394	
ELF3	556902526	0.0109	0.0031	0.9560	
ELF3	556902533	-0.0196	0.0058	0.9394	
ELF3	556902615	0.2580	0.2501	0.6176	
ELF3	556902667	0.7670	2.8000	0.0960	
ELF3	556902676	-0.5451	3.5050	0.0628	
ELF3	556902751	-0.0236	0.0554	0.8142	
PPD-H1	29126139	-0.3034	3.8308	0.0518	
PPD-H1	29126143	0.4809	1.6798	0.1966	
PPD-H1	29126332	0.4810	0.6327	0.4274	
PPD-H1	29126335	-0.2708	1.6508	0.2005	
PPD-H1	29126530	0.2743	2.2701	0.1336	
PPD-H1	29126622	0.3175	0.7773	0.3791	
PPD-H1	29126627	-0.1067	0.1989	0.6561	
PPD-H1	29126640	0.0574	0.1716	0.6791	
PPD-H1	29126657	-0.5611	1.1107	0.2933	
PPD-H1	29126792	-0.0190	0.0301	0.8624	
PPD-H1	29126802	-0.2414	7.0434	0.0087	**
PPD-H1	29126820	-0.0081	0.0157	0.9004	
PPD-H1	29126824	0.7001	3.9791	0.0476	*
PPD-H1	29126843	0.0774	0.1852	0.6675	
PPD-H1	29127002	-0.3712	1.6175	0.2051	
PPD-H1	29127021	0.4579	1.2188	0.2710	
PPD-H1	29127102	0.0411	0.0261	0.8719	
PPD-H1	29127381	-0.0513	0.0492	0.8247	
PPD-H1	29127414	-0.4671	2.1859	0.1410	
CEN4	523378047	0.0173	0.0268	0.8700	
CEN4	523378213	-0.0667	0.0623	0.8032	
CEN4	523378374	0.1002	0.1285	0.7204	
CEN4	523378515	-0.1085	0.2310	0.6313	
CEN4	523378669	0.0399	0.0161	0.8992	
LUX1	692191611	-0.2457	3.5047	0.0628	
LUX1	692191942	0.0509	0.3521	0.5537	
VRN2(SNF2P)	640595614	-0.0389	0.0629	0.8022	
VRN2(SNF2P)	640595615	0.0468	0.2290	0.6329	
VRN2(SNF2P)	640595653	0.2651	0.3068	0.5803	
VRN2(SNF2P)	640595662	-0.1569	0.1572	0.6922	
VRN2(SNF2P)	640595698	-0.3095	0.4470	0.5046	
VRN2(SNF2P)	640595703	-0.0354	0.0043	0.9479	
PHYC	598559120	0.0800	1.2057	0.2736	
PHYC	598560653	-0.0404	0.4266	0.5145	
PHYC	598560884	0.0685	0.7988	0.3726	
VRNH1	599132630	-0.0263	0.1594	0.6902	
VRNH1	599132721	-0.0056	0.0061	0.9376	
CO1	127677476	0.0286	0.1380	0.7107	
CO1	127677509	0.0301	0.2296	0.6324	
CO1	127677980	-0.0403	0.3640	0.5470	
CO1	127678241	0.0307	0.2760	0.5999	
CO1	127678393	0.0552	0.4705	0.4936	
	Constant		0.0335	0.8549	

**,*: Significant at the 1% and 5% levels, respectively.

TRANSPARENT METHODS

Plant materials and growth conditions

A total of 274 accessions (Table **S1**) were selected from the barley worldwide collection preserved at Okayama University (Barley DB: http://earth.nig.ac.jp/~dclust/cgi-bin/index.cgi) to include geographical diversity of collected regions. A set of 274 accessions was sown in fall of 1996–2015; days to heading from sowing (DHS) was scored in the spring of the following years (1997–2016) at the experimental field in Kurashiki, Japan (34°35′N and 133°46′E). Ten plants of each accession were grown in a single row. Rows were 90 cm apart. Within-row spacing was 4 cm. DHS was scored when more than half of the plants in a plot headed.

DNA extraction and marker genotyping

To genotype the 274 accessions, genomic DNA was extracted using a GENE PREP STAR PI-480 (KURABO, Osaka, Japan) according to the manufacturer’s protocol (PLANT version 1). After quality check, DNA samples were genotyped via an Illumina GoldenGate® assay using a 384 single nucleotide polymorphism (SNP) platform developed from BOPA1 (barley oligonucleotide pooled assay 1) based on the genetic map position of Close et al. (2009) to avoid duplicate markers (harvest.ucr.edu). All SNP genotyping data were analyzed using GenomeStudio software (Illumina, USA).

Population structure analysis

The population structure of accessions was estimated from the SNP genotyping data using STRUCTURE version 2.3.4 (Prichard et al., 2000) with an Admixture Model and Markov chain Monte Carlo chain length of 40,000 following a burn-in period of 20,000. Heterozygous alleles identified in the Illumina GoldenGate® assay were eliminated from the analysis. Markers with less than 5% minor allele frequency and genotyping less than 90% of accessions were also eliminated from the analysis. The appropriate number of ancestral population groups (Q) was estimated by calculating ΔK (Evanno et al., 2005). A phylogenetic tree of 274 accessions was reconstructed by the neighbor-joining method in MEGA7 with 1,000 bootstrap replications (Kumar et al., 2016) and visualized on the iTOL web service (https://itol.embl.de/ Letunic and Bork, 2019).

Genotype analysis of genes related to heading

Genomic regions harboring or flanking the eight flowering-related genes or markers *ELF3*, *PPD-H1*, *CEN4*, *LUX1*, *SNF2P* (a marker linked to *VRN2*; Cuesta-Marcos et al., 2010), *PHYC*, *VRN1*, and *CO1* were genotyped in the 274 accessions by PCR amplicon sequencing. Primer sequences are listed in Table **S5**. Multiplexed PCR and preparation of amplicon libraries were performed as described previously (Onda et al., 2018). Briefly, a Multiplex PCR assay kit (version 2; TaKaRa, Kusatsu, Japan) was used with 20 ng of genomic DNA (5 ng/μL) as template and 100 μM of each of the primers. Thermo-cycling conditions comprised preheating at 94°C for 1 min, 30 cycles of 94°C for 30 s and 60°C for 4 min, and a final extension at 72°C for 10 min. Purified PCR products were used for library preparation

with a SPARK DNA sample prep kit for Ion Torrent (Enzymatics, Beverly, MA, USA) and an Ion Xpress Barcode Adapters 1-96 kit (Thermo Fisher Scientific K.K., Japan). The amplicon libraries were sequenced using the Ion Proton System with Ion PI Chip and an Ion PI Hi-Q Sequencing 200 kit (Life Technologies, Japan). The amplicon sequencing reads were mapped to the reference genome sequence of *H. vulgare* cv. Morex (Mascher et al., 2017) retrieved from Phytozome (Hvulgare_462_r1) using the BWA-MEM algorithm of the BWA software (v0.7.17) (Li & Durbin, 2010). Sequence polymorphisms were called using the mpileup command and bcftools of samtools software (0.1.19) (Danecek and McCarthy, 2017).

Regression analysis

Linear regression models of heading date on genetic and environmental factors

A linear regression analysis was conducted to determine the genetic and environmental factors controlling DHS in barley using the lm function of R software (R Core Team, 2020). A dataset comprising 257 accessions without missing values across 20 growing seasons (years) was used to build linear regression models with genetic factors (accession, country of origin, population cluster, spring/ winter growth habit and kernel two/six-row) and an environmental factor (growing season) as the explanatory variables. Days to heading from sowing (DHS) was used as the objective variable. Degree of freedom, adjusted R^2 and F-statistics were calculated by the lm function of R software. Regression models for interactions between genetic and environmental factors were also built.

Linear regression model of heading date on allelic variation of known flowering-related genes

Linear regression models were also built using the lm function of R software to examine allelic combinations of well-known flowering-related genes in barley to estimate DHS. A heading date dataset comprising 234 accessions without missing values for both amplicon sequencing-based genotype data and heading date across 20 seasons was used to build the linear regression models. Based on the amplicon-sequencing-based SNPs, we identified a set of 51 linearly independent SNPs on the genomic regions of eight flowering genes. These SNPs and an environmental factor (growing season) were used as the explanatory variables. DHS was used as the objective variable. The p-value against the null hypothesis the regression coefficient is zero, degree of freedom, adjusted R^2 and F-statistics were calculated by the lm function of R.

Finlay-Wilkinson linear regression analysis of accessions and seasons

To assess phenotypic plasticity of the 257 accessions across different seasons, linear regression of DHS for each accession on means of 20 growing seasons were analyzed by the method of Finlay and Wilkinson (1963) using the lm function of R software. The method of Finlay-Wilkinson measures accession responses to seasons, e.g. accessions with low linear regression coefficients change less DHS but those with high coefficients change more DHS according to the different growing conditions of 20 seasons. The linear regression analysis of Finlay-Wilkinson was also applied to characterize each season on means of 257 accessions to estimate the plasticity of each season. Correlation coefficients between Finlay-Wilkinson linear regression coefficients and other parameters were calculated to estimate contributions of these parameters for the stability of DHS.

Multiple regression analysis of heading response on SNPs in flowering genes

To estimate the contribution of the flowering genes to the heading response of the barley accessions, a multiple regression analysis of the Finlay-Wilkinson linear regression coefficient of DHS (dependent variable) on SNPs in the eight flowering genes (independent variables) was performed using the package BellCurve for Excel.

SUPPLEMENTAL REFERENCES

Arifuzzaman, M., Günal, S., Bungartz, A., Muzammil, S.P., Afsharyan, N., Léon, J., Naz, A.A. (2016) Genetic Mapping Reveals Broader Role of Vrn-H3 Gene in Root and Shoot Development beyond Heading in Barley. PLoS One 11, e0158718.

Danecek, P., and McCarthy, S.A. (2017) BCFtools/csq: haplotype-aware variant consequences. Bioinformatics 33, 2037-2039.

Evanno, G., Regnaut, S., and Goudet, J. (2005) Detecting the number of clusters of individuals using the software STRUCTURE: a simulation study. Mol. Ecol. 14, 2611–2620.

Kumar, S., Stecher, G., and Tamura, K. (2016) MEGA7: Molecular Evolutionary Genetics Analysis Version 7.0 for Bigger Datasets. Mol. Biol. Evol. 33, 1870-1804.

Letunic, I., and Bork, P. (2019) Interactive Tree Of Life (iTOL) v4: recent updates and new developments. Nucl. Acids Res. 47, W256-W259.

Li, H., and Durbin, R. (2010) Fast and accurate long-read alignment with Burrows-Wheeler transform. Bioinformatics 26:589-595.

Onda, Y., Takahagi, K., Shimizu, M., Inoue, K., and Mochida, K. (2018) Multiplex PCR Targeted Amplicon Sequencing (MTA-Seq): Simple, Flexible, and Versatile SNP Genotyping by Highly Multiplexed PCR Amplicon Sequencing. Frontiers Plant Sci. 29, 201.

Pankin, A., Campoli, C., Dong, X., Kilian, B., Sharma, R., Himmelbach, A., Saini, R., Davis, S.J., Stein, N., Schneeberger, K., von Korff, M. (2014) Mapping-by-Sequencing Identifies HvPHYTOCHROME C as a Candidate Gene for the early maturity 5 Locus Modulating the Circadian Clock and Photoperiodic Flowering in Barley. Genetics 198, 383–396.

R Core Team. (2020) R: A language and environment for statistical computing. R Foundation for Statistical Computing, Vienna, Austria. (https://www.R-project.org/)

Stracke, S., Haseneyer, G., Veyrieras, J.B., Geiger, H.H., Sauer, S., Graner, A., Piepho, H.P. (2008) Association mapping reveals gene action and interactions in the determination of flowering time in barley. Theor. Appl. Genet. 118, 259-73.
